# Automatic detection of small lung nodules on CT utilizing a local density maximum algorithm

**DOI:** 10.1120/jacmp.v4i3.2522

**Published:** 2003-06-01

**Authors:** Binsheng Zhao, Gordon Gamsu, Michelle S. Ginsberg, Li Jiang, Lawrence H. Schwartz

**Affiliations:** ^1^ Department of Medical Physics and Department of Radiology Memorial Sloan‐Kettering Cancer Center 1275 York Avenue New York New York 10021; ^2^ Department of Radiology Weill Medical College of Cornell University New York New York 10021; ^3^ Department of Radiology Memorial Sloan‐Kettering Cancer Center 1275 York Avenue New York New York 10021

**Keywords:** computer‐aided detection (CAD), computed tomography, lung nodule, image processing, local density maximum

## Abstract

Increasingly, computed tomography (CT) offers higher resolution and faster acquisition times. This has resulted in the opportunity to detect small lung nodules, which may represent lung cancers at earlier and potentially more curable stages. However, in the current clinical practice, hundreds of such thin‐sectional CT images are generated for each patient and are evaluated by a radiologist in the traditional sense of looking at each image in the axial mode. This results in the potential to miss small nodules and thus potentially miss a cancer. In this paper, we present a computerized method for automated identification of small lung nodules on multislice CT (MSCT) images. The method consists of three steps: (i) separation of the lungs from the other anatomic structures, (ii) detection of nodule candidates in the extracted lungs, and (iii) reduction of false‐positives among the detected nodule candidates. A three‐dimensional lung mask can be extracted by analyzing density histogram of volumetric chest images followed by a morphological operation. Higher density structures including nodules scattered throughout the lungs can be identified by using a local density maximum algorithm. Information about nodules such as size and compact shape are then incorporated into the algorithm to reduce the detected nodule candidates which are not likely to be nodules. The method was applied to the detection of computer simulated small lung nodules (2 to 7 mm in diameter) and achieved a sensitivity of 84.2% with, on average, five false‐positive results per scan. The preliminary results demonstrate the potential of this technique for assisting the detection of small nodules from chest MSCT images.

PACS number(s): 87.57.–s, 87.90.+y

## INTRODUCTION

Lung cancer is the leading cause of cancer death in both men and women in the USA. In 2002, it is estimated that there would be 169 400 newly diagnosed cases of lung cancer and 157 900 deaths from this disease in the United States.1 More people die of lung cancer than of colon, breast, and prostate cancers (the next three most deadly cancers) combined. Although surgery, radiation therapy, and chemotherapy have been used in the treatment of lung carcinoma, the five‐year survival rate for all stages combined is only 14%. This has not changed in the past three decades. It is reported that the survival rate for localized cancer (stage I) is 49%. However, only 15% of lung cancers can be discovered that early.^2^ Assuming that intervention at early stages leads to higher survival rates, it is thus a major public health directive to improve the survival rate and to reduce the mortality of lung cancer through detection and intervention at an earlier and potentially more curable stage.

CT is considered to be the most accurate imaging modality available for early detection and diagnosis of lung cancer.^3–^, ^8^ Multislice CT (MSCT), utilizing multiple detector row technology, has increased scanning speed. As a consequence, volumetric CT chest images can be acquired with a single breath hold, with 1–3 mm axial collimation These thin sections can facilitate the detection of small nodules that may include lung cancers at early stages. However, hundreds of CT images per examination are difficult to interpret in the traditional axial mode, leading to a high false‐negative rate for detecting small nodules. The reasons are multiple and are related to the conspicuity of nodule itself (size, density, and location), human error, and scanning technique (e.g., radiation dose, slice thickness).

Although CT may be capable of depicting lung nodules as small as 1 mm,^9^ a three‐dimensional (3D) computer simulated nodule study demonstrated the overall detection rate to be only 63% for nodules 1–7 mm in diameter. As the size of the nodule decreased, the sensitivity fell and only 48% of nodules less than 3 mm and 1% of nodules less than 1.5 mm were detected.^10^ Furthermore, retrospective analysis of CT scans constantly revealed undetected lung cancers on the initial scans.^11–^, ^13^

Manipulation of volumetric CT data sets may improve a radiologist's ability to detect small lung nodules. For example, reconstruction of CT images with narrow interscan spacing^14^ and interpretation of images using cine rather than film‐based viewing technique^15,^, ^16^ have been reported to improve small nodule detection. With the fast advancement of computer software and hardware, there is an urgent need to develop computer‐assisted tools for the optimized detection and quantitative evaluation of the large number of small nodules identified by volumetric chest CT in both diagnostic and screening studies.

A number of computer‐aided methods and systems for the automated detection of small nodules from CT chest images have been developed over the years.^17–^, ^26^ From the technical point of view, they can be divided into two groups of approaches: density‐based and model‐based approaches. Considering the fact that lung nodules have relatively higher densities than those of lung parenchyma, density‐based detection methods employ techniques such as multiple thresholding,^8,^, ^17,^, ^18^ region‐growing,^19^ locally adaptive thresholding in combination with region‐growing,^20^ and fuzzy clustering^21^ to identify nodule candidates in the lungs. False‐positive results can then be reduced from the detected nodule candidates by employing *a priori* knowledge of small lung nodules. For the model‐based detection approaches, the relatively compact shape of a small lung nodule is taken into account while establishing the models to identify nodules in the lungs. Techniques such as “N‐Quoit filter,”^22^ template‐matching,^23^ object‐based deformation,^24^ and the anatomy‐based generic model^25^ have been proposed to identify sphere‐shaped small nodules in the lungs. Other attempts include automated detection of lung nodules by analysis of curved surface morphology^26^ and improvement of the nodule detection by subtracting bronchovascular structures from the lung images.^27^ Due to the relatively small size of the existing CT lung nodule databases and the various CT imaging acquisition protocols, it is hard to compare the detection performance among the developed algorithms.

Because of the wide range of density distribution of lung nodules on CT images, the multiple thresholding technique in combination with the feature extraction and classification appears to be a practical approach to effective and efficient nodule detection. In this paper, we present our preliminary study on the development of an advanced multiple thresholding method for the automated detection of small lung nodules. The method uses a three‐step approach. The first step is to automatically extract the lungs from MSCT images by analyzing the volumetric density histogram, thresholding the original images, and subsequently applying a morphological operation to the resultant images. The second step is to identify higher density structures (e.g., nodules, vessels) spread throughout the extracted lungs using a local density maximum (LDM) algorithm. The last step is to reduce false‐positive results from the detected nodule candidates using *a priori* knowledge of the lung nodules. The detection method has been validated with computer simulated small lung nodules.

## METHODS

## A. Automated extraction of the lungs

**Figure 1:**
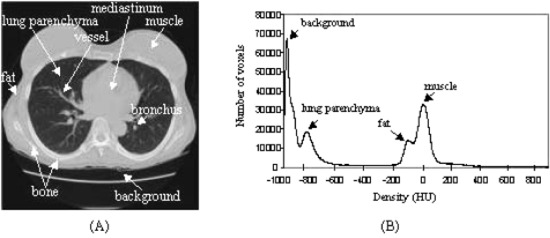
(Color) A single chest CT slice and the corresponding volumetric density histogram. (a) Anatomic structures of the lungs are marked on the CT image. (b) A typical volumetric chest CT histogram with four density frequency‐peaks on it.

The basis of the lung segmentation involves finding a threshold in the density histogram of CT chest images. One 3.75‐mm‐thick slice from a CT chest series (Lightspeed QX/i, GE Medical Systems, Milwaukee, WI) and the corresponding volumetric density histogram are shown in Fig. 1. Typically, there are four peaks on the histogram, representing, from left to right, background outside the body, lung parenchyma, fat, and muscle, respectively. Bones have higher density than those of muscles but they have too few voxels to form a peak. A long, flat, and low valley between the peaks of the lung parenchyma (second peak from the left) and the fat (third peak from the left) on the histogram indicates that the separation of the lung parenchyma from the soft tissues (fat and muscles) and bones is insensitive to the density threshold set within the valley. A threshold thus lying in the lung parenchyma‐fat valley can be chosen after automatic determination of the high peaks. This threshold is then used to initially separate the lung parenchyma from the other anatomic structures on the CT images.

**Figure 2:**
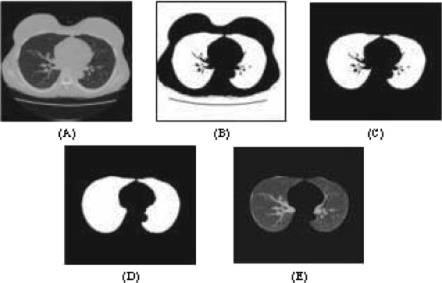
(Color) Automatic extraction of the lungs from chest CT images. (*A*) Original image. (b) Thresholded image. Threshold level=−375 HU. (c) Initial lung mask. (d) Complete lung mask. (e) Extracted lungs.

Voxels having a density lower than the threshold value will be recognized as lung candidates and assigned the value of 1 and appear white in Fig. 2(b), whereas other voxels are assigned the value of 0 and appear black in Fig. 2(b). Due to their low densities, both the lung parenchyma and background will be classified as the “lung” on the resultant binary images. As the lung parenchyma is usually completely isolated from the background by the chest wall, it can be readily determined by labeling 3D connected components (i.e., grouping geometrically connected voxels that have the value of 1 and assigning an identical number to the voxels in each individual group) and selecting the largest component that does not touch any margin of the images [Fig. 2(c)]. As the apparent density of vessels and bronchial walls in the lung differ, structures with higher densities including some higher density nodules could be grouped into soft tissues and bones, leading to an incomplete extraction of the lung mask [Fig. 2(c)]. To obtain a complete, hollow‐free lung mask, morphological closing is applied [Fig. 2(d)]. Spherical shape of the structural element is chosen for the morphological operator and the filter size is experimentally determined. With the 3D mask, the lungs can be readily extracted from the original chest CT images [Fig. 2(e)].

In the image processing steps gray level values rather than Hounsfield units are utilized. All values of gray level obtained from the GE Lightspeed QX/i machine equate to Hounsfield units HU as follows: gray level=HU+1024.

## B. Detection of nodule candidates using a local density maximum algorithm

Blood vessels, bronchial walls, and nodules have density values higher than those of the lung parenchyma on CT images. A 3D algorithm, local density maximum (LDM), has been thus developed for locating those higher density structures scattered throughout the lungs.

**Figure 3:**
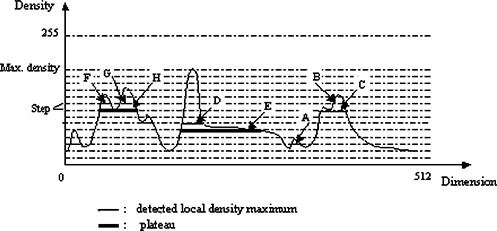
(Color) One‐dimensional example of the detection of local maximum. Detected local maxima are marked with a thin line and plateaus with a thick line. *A* is a newly detected object; *C* (local maximum) replaces *B; D* is a local maximum, whereas *E* is a plateau; *F* and *G* are two local maxima residing at the plateau *H*.

The algorithm can be intuitively explained with a one‐dimensional example. Suppose the curve in Fig. 3 is a density profile. The LDM algorithm begins to threshold it with an initial threshold value that can be the maximal density value of the profile. Objects (an object is a group of connected points with density values greater than the threshold) can be identified through labeling connected components. Subsequently, the threshold level decreases in a stepwise manner and as a result more objects are identified. Local density maxima (local maxima) can be determined by testing geometric overlap of the objects identified at the current threshold level with the ones detected at the previous level. The detail of the LDM algorithm is given in the appendix.

Three‐dimensional objects are determined at each threshold level by labeling 3D connected components. Geometrical overlapping of the objects identified at the consecutive threshold levels are tested along each of the three directions (i.e., *x, y*, and *z* directions), respectively.

Five parameters (i.e., the threshold step, threshold stop value, minimal density peak of local maximum, minimal size of local maximum, and the ratio specifying the change of object's volume to its surrounding box's volume) are used in the algorithm. The minimal size is chosen based on the size range of nodule of interest as well as the CT in‐plane resolution. The other parameters control the density difference between a local maximum and its background. All parameters are determined experimentally. In this work, we chose 7 (gray level) as the threshold step, 15 (gray value) as the threshold stop value, 12 (gray level) as the density peak, 10 (voxel) as the minimal size, and 1/30 as the ratio *r*.

## C. Reduction of false‐positive results

Local maxima identified by the LDM may have any size and shape as the information about the nodule size (except the smallest size) and shape are not integrated into the algorithm. The next step is, therefore, to apply the *a priori* knowledge of small lung nodules to eliminate those local maxima which are not likely to be lung nodules. Such information includes, but is not limited to, the size and shape of lung nodules that need to be detected. Only those that fall into the size range of nodule of interest will be reserved for further consideration as nodules. In addition, although nodules on a CT slice may have similar appearance to blood vessels that run through the plane perpendicularly, nodules and vessels have very distinct shapes in three dimensions. The former have a near spherical shape, whereas the latter have a tubular shape. The following parameters are thus defined in this work to distinguish nodules from non‐nodules.

**Figure 4:**
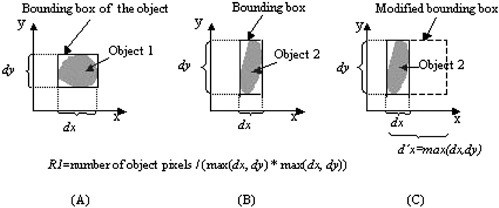
(Color) Explanation of the modification of an object's bounding box in two dimensions. Objects in (a) and (b) have very distinct shapes. If we use the bounding box to define the parameter of *R*1, i.e., R1=number of object pixels/(dx*dy), the two objects will have similar values of *R*1. However, If we use a modified bounding box (*c*) to define the parameter of *R*1, i.e., R1=number of object pixels/[max(dx,dy)][max(dx,dy)], the two objects will have distinct values of *R*1.

(1) *R* 1 is defined as the ratio of the volume of the object to the volume of a modified bounding box of the object, i.e.,

(1) R1=number of object voxels/{[max(dx,dy)][max(dx,dy)]dz},

where, *dx, dy*, and *dz* are the maximal projection lengths (in pixel) of the object along the axes of *x, y*, and *z*, respectively, and max(dx,dy)=dx, if dx≥dy; otherwise, max(dx,dy)=dy. The volume of the box containing an object is given by the denominator in Eq. (1) rather than by dx⋅dy⋅dz. This allows *R*1 to be able to distinguish between a compact‐shaped object from a sticklike object lying in the lungs at a small angle to either the *x* or *y* coordinate axis. Figure 4 explains the modification of the bounding box of an object in two dimensions.

(2) *R*2 is defined as the ratio of the maximal projection length of the object along the axis of *z* to the maximal projection length of the object along the axis of *x* or *y*, whichever is larger. The nonisotropic characteristics of CT scan are taken into account by multiplying the length (in pixel) with the corresponding pixel size (in mm).

(2) $$R2=(\mathrm{dz})p-{\text{size}}_{z}/\left\{\left[\text{max}(\mathrm{dx},\mathrm{dy})\right]p-{\text{size}}_{x}\right\},$$

where $p-{\text{size}}_{z}$ and $p-{\text{size}}_{x}$ are the pixel size in *z* and *x(y)* directions, respectively.

(3) *R*3 is defined as the ratio of the maximal projection length of the object along the axis of *x* or *y*, whichever is larger to the maximal projection length of the object along the axis of *x* or *y*, whichever is smaller.

(3) R3=max(dx,dy)/min(dx,dy),

where min(dx,dy)=dx, if dx<dy; otherwise, min(dx,dy)=dy.

An object (nodule candidate) will be considered as noncompact or not within the size range of interest, and thus be deleted if the following expression is true:

(4) $$\begin{array}{l} R1<0.3||R2>5.0||R2<0.2||R3>1.5||\{\left[(\mathrm{dx})p-{\text{size}}_{x}<2.0\right]\\ \;\;\;\;\;\;\;\;\;\;\&\&[\left(\mathrm{dy})p-{\text{size}}_{y}<2.0\right]\}||\left\{\left[(\mathrm{dx})p-size_{x}>15.0\right]\&\&\left[(\mathrm{dy})p-{\text{size}}_{y}>15.0\right]\right\}, \end{array}$$

where || is the logical or and & is the logical and. The threshold values for *R*1, *R*2, and *R*3 are determined experimentally.

The above‐defined parameters and conditions ensure that the remaining objects have relatively compact shapes in both three dimensions (*R*1) and two dimensions (*R*2 and *R*3) and fall into the size range of nodule. Although nonisotropic characteristics of CT scanner is taken into account while calculating *R*2, the threshold level of *R*2 is deviated from 1 (the closer the value of *R*2 to 1, the more compact shape an object will possess). This is because the partial volume artifact along *z* axis is larger than that along *x* and *y* axes.

## D. Nodule simulation

**Figure 5:**
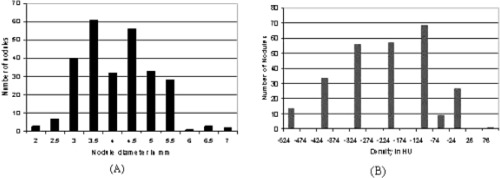
(Color) Size and density distributions of the computer simulated lung nodules. (a) Nodule size distribution (mean: 4.1 mm; std: 0.9 mm). (b) Nodule density distribution (mean: mean: −233.1  HU; std: 140.7 HU).

A computer simulation program generated a bank of lung nodules which were small spheres and ellipsoids of various sizes and densities. The size of nodules varied in diameter from 2 to 7 mm (mean: 4.1 mm; std: 0.9 mm); densities were between the average densities of the lung parenchyma and soft tissues (mean: −233.1 HU; std:140.7 HU); and shapes were sphere and ellipsoid (the ratio of the long axis to the short one of an ellipsoid being <1.5). Figure 5 shows the size and density distributions of the simulated nodules.

Before adding the nodules onto CT images, the nodule images (a nodule image only contains the nodule) were smoothed with a 3D Gaussian low‐pass filter. Visually, we found that Gaussian filter with a length of 5 voxels produced the most similar edge appearance of the simulated nodules to those of surrounding vessels that are perpendicular to the plane of the CT images. Due to the nonisotropic resolutions of CT images, the chest images were first interpolated into an isotropic scale. Nodules were then added onto those isotropic images. The chest images containing simulated small nodules were then subsampled back to the original resolutions before the nodule detection algorithm was applied.

Thirty to thirty‐five such smoothed small nodules were then added onto each of eight normal noncontrast chest examinations. The protocol of these clinically acquired diagnostic images was: 120/140 kVp, 220/290 mAs, pitch of 3:1, and 3.75 mm collimation/3.75 reconstruction interval. Nodules were added throughout various parts of the lung fields, including in some cases, areas in which they were “attached” to vessels. Geometrically, 20% of the nodules were distributed in the peripheral zone of the lungs (within approximately 2 cm of the pleural surfaces), 7% in the central zone (within approximately 2 cm of the mediastinum), and 73% in the middle zone (between the peripheral and the central). To simulate clinical practice with lung cancers or lung metastases, particular attention was paid so that small and low density nodules were placed in the peripheral zone of the lungs and nodules that imitated the surrounding vessels running perpendicularly through the CT planes were placed in the central and the middle zones of the lungs. Furthermore, nodule densities, in general, should not be greater than those of adjacent vessels.

## RESULTS

In total, 266 simulated small nodules were added onto eight normal chest CT scans, each scan having 60 to 80 slices. 251 nodules were detected by the LDM algorithm, corresponding to a detection sensitivity of 94.4%. There were 906 false‐positive nodules per case. After applying the algorithm for the reduction of false‐positive results, 224 nodules were retained, corresponding to a final detection sensitivity of 84.2%. The average number of false‐positives per case was reduced to only 5 (ranged 1 to 9). Furthermore, 11.4% of the nodules placed in the central zone, 22.2% of the nodules placed in the peripheral zone, and 15.8% of the nodules placed in the middle zone of the lungs were not detected by the algorithm. Among them $f23 were initially detected by the LDM as local maxima (nodule candidates) but then deleted by the reduction of false‐positives in the next step.

**Figure 6:**
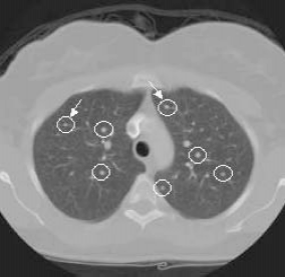
(Color) Small nodule detection using the LDM algorithm. All inserted seven nodules were successfully detected (circles), including the two that were attached to vessels (arrows).

**Figure 7:**
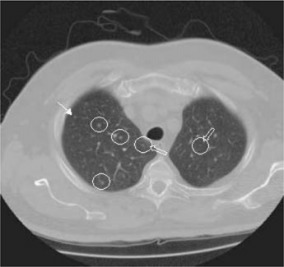
(Color) Small nodule detection using the LDM algorithm with two false‐positive results. Three of the four inserted nodules were detected (circles), one was missed (arrow), and there were two falsely detected nodules (circles and open arrows).

Figures 6 and 7 show representative CT images with computer simulated nodules and the corresponding results to demonstrate the ability of the detection algorithm. Figure 6 presents an example where seven small nodules were added onto the image. The algorithm successfully detected all of them (circle), including the two that were attached to vessels (arrows). There was no false‐positive result on the image. Figure 7 shows another example with false‐positive results and missed nodules. Four small nodules were added onto the image. Two of them were placed to imitate surrounding blood vessels that ran perpendicularly through the CT plane, and the other two small and low‐density nodules were placed in the peripheral zone of the lung. Three of the four added nodules were detected (circle), one was missed (arrow), and there were two falsely detected nodules (circle and open arrow).

**Figure 8:**
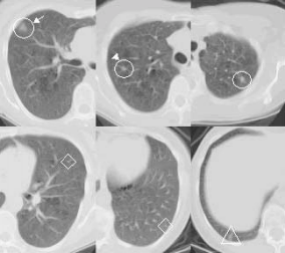
(Color) Application of the detection algorithm to a clinical case with actual lung nodules. Three of the four nodules were detected (circles), including one ground‐glass opacity nodule (circle and arrow) and one nodule that was attached to the surrounding vessels (circle and arrow head). One nodule was not detected (triangle). There were nine false‐positives in total and two of them are shown as examples (diamonds).

The algorithm, without any modification, was directly applied to a clinical subject with four actual small lung nodules. The imaging protocol was different than the one used in the simulation study, i.e., 120 kVp, 150 mAs, 7.0 mm collimation and 7.0 reconstruction interval. Figure 8 shows the detection result. Three of the four nodules were successfully detected by the algorithm (circle), including one ground‐glass opacity nodule (circle and arrow) and one nodule that was attached to the surrounding vessels (circle and arrow head). There were nine false‐positive results, and two of them are shown in Fig. 8 (diamonds). One nodule was not detected (triangle).

## DISCUSSION

The essence of our detection method lies in its ability to automatically search for higher density structures including nodules scattered in the lungs through sequentially declining threshold level. This search of higher density structures can be terminated locally if any of the termination criteria of identifying local maxima is met. Nodules having a wide range of density distribution can thus be automatically identified from both homogeneous and heterogeneous lung parenchyma as the plateau in which a nodule resides is defined locally. Furthermore, nodules that attach to adjacent vessels can also be identified, as long as their densities are different to those of the vessels at the contact points by a certain amount.

One limitation of a region‐growing‐based technique is that it may not detect lower density nodules if seed points from where suspicious nodule regions start to grow do not include any part of those lower density nodules. Difficulties may also rise with a cluster‐based technique when dealing with the lung nodules of a wide range of densities, i.e., of mixed attenuation. The nodules may be falsely classified as blood vessels (for those nodules having higher density values) or as lung parenchyma (for those nodules having lower density values). One of the advantages of our method over the existing multiple thresholding detection technique is that objects detected at different threshold levels can be efficiently tracked and merged to a single one if they belong to each other. This is not possible with the existing technique,^8,^, ^17,^, ^18^ where objects detected at each given threshold level are all considered as nodule candidates and are subjected to the subsequent feature extraction and classification for the reduction of false‐positives. There exists a data redundancy in the feature extraction and classification of the existing technique as an object detected at a higher threshold level belongs to a part of the object detected at a lower threshold level, if they geometrically overlap with each other. This redundancy will cause unnecessary computation in the feature extraction and may create confusion in the classification. In our approach, the data redundancy can be avoided by identifying and merging identical objects detected at different thresholds.

Potentially, there are two parts in the algorithm where the detection sensitivity and the rate of false‐positives may be affected. They are the identification of local maxima and the reduction of false‐positives. To obtain a higher detection sensitivity and lower rate of false‐positive results, lung nodules should be identified as local maxima (nodule candidates) as many as possible, while the effects of heterogeneity of lung parenchyma and vascular structures as well as noise should be maximally suppressed. This is because if a nodule is not detected as a local maximum by the LDM algorithm, it will become a false‐negative. On the other hand, all non‐nodule local maxima that are not able to be eliminated by the reduction of false‐positives will become false‐positives. Even if a nodule is recognized as a local maximum by the LDM algorithm, it may still be incorrectly ruled out in the process of the reduction of false‐positives (i.e., may also become a false‐negative). Obviously, in the reduction of false‐positives among the detected nodule candidates there is a balance between preserving nodules and eliminating non‐nodules using the extracted features. By cautiously adjusting the algorithm's parameters, a satisfactory detection performance can be achieved. In our simulation study, a detection sensitivity of 94.4% was reached with the LDM algorithm (i.e., 94.4% of nodules were detected as local maxima). However, a total number of detected nodule candidates could be larger than 1000. After applying the extracted features to reduce false‐positives, the number of false‐positives per case was reduced to 1–9 (average: 5). However, the detection sensitivity dropped to 84.2%. It was reported in the Results section that two thirds of false‐negatives (missed nodules) were initially detected by the LDM as local maxima. They were deleted later by the reduction of false‐positives. This indicates that the detection sensitivity may be increased by improving the strategies for the reduction of false‐positives. The remaining false‐positives are mainly caused by the heterogeneous density of vessels due to the partial volume artifact of CT scanners. Those segments of vessels appearing with higher densities and possessing compact shapes may be recognized as nodule candidates. They cannot be simply ruled out by using the extracted features, since they share common characteristics with nodules. However, they may be removed by including additional information about, for example, plateau's density into the detection algorithm.

Unlike the radiologist's detection of small lung nodules, vessels that are perpendicular to the CT planes (i.e., they have nodulelike appearances in the plane) should not mislead the computer interpretation. This is because the detection algorithm can distinguish between a spherical small nodule and a cylinder vascular structure by analyzing the 3D shapes. Theoretically, the performance of a computer‐aided detection method should not be affected by the nodule location as long as the nodules are not attached to the surrounding structures that have identical or similar density distributions to those of the nodules. In our simulation study, the missing rates of the nodules placed in the central and middle zones of the lungs were similar (11.4% versus 15.8%). Small and low density nodules were placed in the peripheral zone of the lungs, accounting for the higher false‐negative rate of the peripheral nodules (22.2%). This may be improved by adapting the feature thresholds in the reduction of false‐positives.

In this preliminary study, computer simulated nodules were added onto clinically acquired CT chest images to demonstrate the ability of our algorithm to detect small lung nodules. The simulated nodules were created with clinically relevant sizes and densities, and were placed throughout the lungs. Unlike evaluation of the visual detection performance where simulated nodules are strictly required to imitate real nodules, particularly to imitate nodule edge appearances, evaluation of the performance of computer‐aided detection algorithms may not require such strictly similarities between real and simulated nodules. For the detection algorithm developed in this work, factors that are related to the nodules and may affect the algorithm's performance are the density differences between a nodule and its surrounding background and the nodule shape. The algorithm is thus sensitive to image random noise caused by quantum mottle and structural distortions brought in by the CT reconstruction algorithm as they may change the nodule‐lung parenchyma relationship. In the simulation study, random noise and structural distortions were unchanged in the images containing the computer‐generated nodules as the nodules were simply added onto the images. Therefore, the parameters of the LDM algorithm may not necessarily need to be modified when the algorithm is transferred from the simulation study to clinical studies. With regard to the nodule shape, sphere and ellipsoid were used to model the lung nodules in the simulation study since small lung nodules tend to be compact. Although the nodule shape has little effect to the LDM algorithm, it does influence the performance of the reduction of the false‐positives because the latter utilizes the shape features to distinguish between nodules and non‐nodules. In our previous study on the segmentation of small lung nodules on volumetric CT images, we found that the 3D shape compactness factor can be sensitive to the object (nodule) size, i.e., the value of the compactness factor may become meaningless for small objects.^28^ This is because the fraction of the number of object surface voxels to the number of entire object voxels increases as the size of an object decreases. The object surface voxels are the voxels that cause errors in the estimation of the 3D compactness factor. Instead of using the 3D shape compactness factor, we defined several 2D and 3D ratios derived directly from *dx, dy*, and *dz*, the maximal projection lengths of an object along the axes of *x, y*, and *z*. We found these ratios practical and useful in the discrimination of small nodules from non‐nodules. Another reason for employing these features is that the values of the features are already available from the output of the LDM algorithm, there is no need to calculate them in the feature extraction. However, in reality, nodules may not be spheres or ellipsoids. Therefore, the features and the threshold levels set for discriminating nodules from non‐nodules in the simulation study may need to be modified or improved so that the detection algorithm can work appropriately for clinical studies.

Nevertheless, without making any changes to the algorithm, a similar result of the detection sensitivity and false‐positive rate was obtained when the detection algorithm was applied to a clinical case with actual lung nodules. It is worth mentioning that the acquisition protocol of the images containing the actual lung nodules was different from the one used in the simulation study. Particularly, the images were acquired with different slice thicknesses (simulation images versus clinical case: 3.75 mm versus 7.0 mm). In addition, the detected actual nodules all had speculated shapes, including a ground‐glass opacity nodule and a nodule that was attached to the surrounding vessels. One nodule was not detected because only small part of it was detected as a nodule candidate by the LDM algorithm. It was then eliminated in the process of reducing false‐positives, as it did not meet the size criterion of being a nodule.

To achieve high detection sensitivity and, in the meanwhile, to keep the number of false‐positives as low as possible, modifications or even redefinitions of the parameters used in the detection algorithm are expected when the algorithm is further validated with a large clinical data set, particularly when the image data sets are acquired under different imaging protocols. Nevertheless, there is an inevitable dilemma, as a high detection sensitivity can be only achieved at the cost of an increasing number of false‐positive results.

The LDM algorithm is designed to detect solid lung nodules on CT images. It should also work for part‐solid nodules as long as the solid part(s) of the nodule exceeds a certain size. It may fail to detect nonsolid nodules. Furthermore, if a nodule is attached to a blood vessel and there is no density difference between the nodule and the vessel at their contact points, the nodule may not be able to be detected by this algorithm. This requires further improvement of the algorithm by developing additional features that allow to identify a compact part (i.e., nodule) from an object (e.g., vessel with a nodule attached).

## CONCLUSION

We have developed an advanced computerized method for the automated detection of small nodules on chest MSCT images. This method uses a three‐step approach, consisting of automatic extraction of the lungs, detection of higher density structures in the extracted lungs, and elimination of false‐positive results among the detected nodule candidates. The method has been validated with computer generated small lung nodules (2 to 7 mm) and achieved a detection sensitivity of 84.2%. There were, on average, five false‐positive nodules per case. Our preliminary study shows the potential of the method for assisting the detection of small lung nodules on MSCT images.

## THE LDM ALGORITHM

The detail of searching for local maxima (nodule candidates) is described in the following. Suppose that ${\text{obj}}_{i}^{c},\;i=1,\ldots n_{c}$ are the objects detected at the current threshold level, and ${\text{obj}}_{i}^{p},\;j=1,\ldots n_{p}$ are the objects detected at the previous threshold level. If for a given object *i* detected at the current threshold level there exists *m* objects detected at the previous threshold level such that ${\text{obj}}_{i}^{c}⫆{\text{obj}}_{k_{l}}^{p};\;k_{l}\leq n_{p}$; l=0,1,…,m; $i=1,\ldots ,n_{c}$, then the following three situations need to be considered.

(i) If m=0, i.e., the current object has no overlap with any of the previous objects. The current object is thus regarded as a new object detected at the current threshold level (e.g., object *A* in Fig. 3).
(ii) If m=0, which specifies that the current object overlaps only one of the previous objects; this object will replace the previous one (e.g., object *C* replaces object *B*). However, there is one exception. We define a ratio parameter r=r1/r2, where *r*1 is the ratio of the volume of the current object to that of its bounding box and *r*2 is the ratio of the volume of the previous object to that of its bounding box. If the ratio *r* drops drastically, the current object will be interpreted as a plateau (object *E*) on which the previously detected one (*D*) will be identified as a local maximum if it meets the following two criteria for being a local maximum. Explicitly, its size and density must be larger than a predefined minimal size and minimal density peak height correspondingly.
(iii) If m>1, which indicates that the current object overlaps more than one previous object; should any of the previous objects meet the criteria of being a local maximum, it will be recognized as a local maximum (*F* and *G*) and the current object becomes a plateau (*H*). Otherwise, it will be treated either as in case (ii) if only one of the previous objects meets the criteria of being a local maximum or as in case (i) if none of the previous objects meets the criteria.

The process of searching for local maxima terminates when the threshold reaches the minimal density value of the lung images, or a predefined density value that can be derived from a statistical analysis of lung parenchyma.
